# Simultaneously Detecting the Power and Temperature of a Microwave Sensor via the Quantum Technique

**DOI:** 10.3390/mi15111305

**Published:** 2024-10-28

**Authors:** Zhenrong Zhang, Yuchong Jin, Jun Tang, Jun Liu

**Affiliations:** 1 Key Laboratory of Instrumentation Science and Dynamic Measurement, School of Instrument and Electronics, North University of China, Taiyuan 030051, China; zhangzr717@163.com (Z.Z.); sz202206093@st.nuc.edu.cn (Y.J.); 2 Key Laboratory of Instrumentation Science and Dynamic Measurement, School of Semiconductor and Physics, North University of China, Taiyuan 030051, China

**Keywords:** NV centers, power detection, temperature detection

## Abstract

This study introduces a novel method for the simultaneous detection of microwave sensor power and temperature, leveraging nitrogen-vacancy (NV) centers as a robust quantum system. Through precise measurement of the optical detection magnetic resonance contrast in NV centers, the microwave power is accurately determined. Furthermore, the temperature of the sensor is obtained by monitoring the variations in zero-field splitting and thorough spectral analysis. This method enables the efficient real-time acquisition of synchronized data on both microwave power and temperature from the sensor, facilitating concurrent monitoring without the necessity of additional sensing devices. Finally, we verified that the magnetic sensitivity of the system is approximately 1.2 nT/Hz^1/2^, and the temperature sensitivity is around 0.38 mK/Hz^1/2^. The minimum resolution of microwave power is about 20 nW. The experimental results demonstrate that this quantum measurement technique provides stable and accurate data across a wide range of microwave power and temperature conditions. These findings indicate substantial potential for this technique in advanced applications such as aerospace, medical diagnostics, and high-frequency communications. Future studies will aim to extend the industrial applicability of this method by refining quantum control techniques within NV center systems.

## 1. Introduction

The progression of microwave technology is crucial for the development of high-performance systems, including radar and satellite communications [1,2,3]. In these applications, the real-time monitoring of microwave devices is vital to guarantee both reliability and safety. This is particularly important in the context of 5G and future communication systems, where microwave components are integral. Effective thermal management is essential for sustaining device performance and prolonging the lifespan of these components [4,5,6]. Continuous monitoring of heating conditions and variations in output power in these devices can prevent performance deterioration or damage caused by overheating, thereby maintaining the stability and efficiency of communication systems [7,8]. A thorough understanding of the relationship between output power fluctuations and thermal conditions is key to optimizing microwave device design, ensuring that optimal performance is maintained under diverse operating conditions [9,10]. This optimization not only improves efficiency but also reduces energy usage, underscoring the considerable theoretical and practical importance of developing reliable methods for microwave power and temperature detection.

Existing technologies for detecting microwave power include thermocouple-based microwave power sensors [11,12], terminal devices utilizing MEMS [13,14], and microwave power sensors, typically categorized into direct heating and indirect heating types [15]. Terminal equipment offers several benefits, such as a low reflection coefficient, high sensitivity, and superior linearity. However, its application is significantly restricted due to low overload power, and the signal becomes unusable after power detection. Current approaches for detecting microwave power involve the use of microwave probes, fiber optic detection, and vector network analyzers. While each method has distinct advantages, they also possess notable limitations. For instance, probe detection provides high resolution but can disrupt the circuit being measured and demands precise operation; fiber optic detection, though less prone to electromagnetic interference, is associated with complex systems and high costs; and vector network analyzers, while highly capable, are expensive and require extensive maintenance. Moreover, there is an urgent demand for a testing system that can quickly and simultaneously acquire both power and temperature data from microwave sensors.

To address these challenges, this study investigates the application of diamond nitrogen-vacancy (NV) center sensors as core sensing units for the real-time measurement of multiple physical parameters in microwave sensors [16]. NV centers are characterized by their exceptional sensitivity and resolution, efficient operation at room temperature, and resistance to electromagnetic interference [17]. These attributes make NV center sensors particularly well suited for the real-time monitoring of high-performance microwave systems. The primary aim of this research is to demonstrate that NV center-based sensors offer an effective and reliable solution for the simultaneous detection of temperature and microwave radiation power. This paper examines the use of NV center sensors in monitoring the power and temperature of microwave devices and proposes optimized design strategies to achieve efficient thermal management and enhance device performance.

## 2. Theory and Experimental Setup

**Figure 1:**
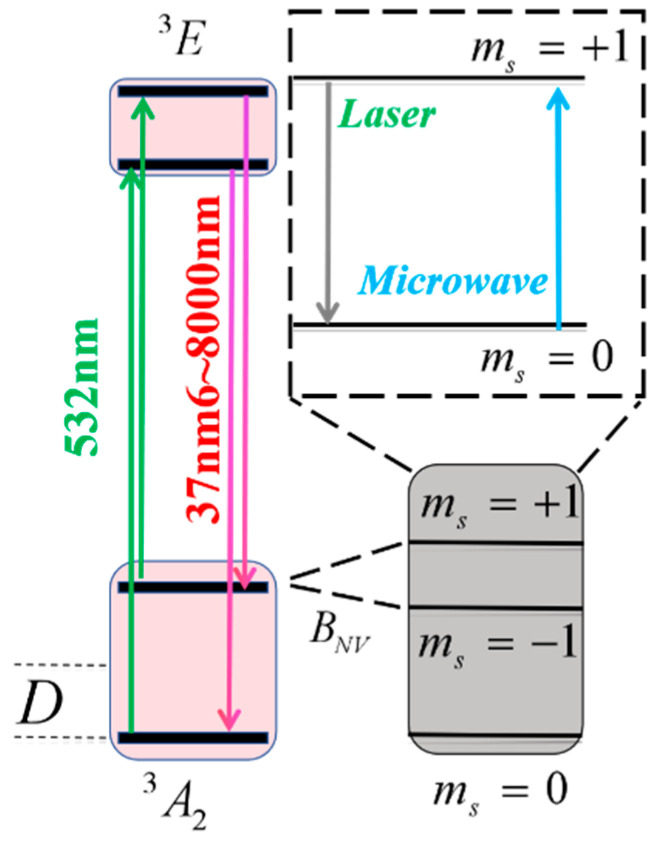
The energy level structure of the NV centers.

As shown in Figure 1, the electron of the ground state has three states: m_s_ = 0, m_s_ = 1, and m_s_ = −1. The energy between m_s_ = 1 and m_s_ = −1 is about 1.945 eV, the zero-field splitting *D* = 2.87 GHz; this value can be changed magnetically because the Zeeman split [18] and the resonance frequency (*f*_NV_) of the m_s_ = ±1 states is *f*_NV_ = *D* ± gB. When subjected to optical pumping, the ground-state spin of the NV center can be initialized to the m_s_ = 0 state. The NV Hamiltonian demonstrates high sensitivity to various physical parameters, and the application of optically detected magnetic resonance (ODMR) allows for straightforward measurement of the spin levels of NV centers [19,20,21]. In the presence of an external magnetic field, the Hamiltonian for the ground-state spin of the NV center is given as follows [22]:

(1) $$H_{\mathrm{NV}}=D(\hat{n}\cdot \vec{S})+g\mu_{B}B$$

where *g* = 28,000 MHz/T is the gyromagnetic ratio, *B* is the external magnetic field along the [001] crystallographic region, and *µ*_B_ is the Bohr magneton. When pumped with a 532 nm laser, the electron spins jump from the ground state at m_s_ = 0 to the excited state at m_s_ = ±1, then decay back to the ground state and emit a fluorescence photon.

In contrast, when the electron spin is in the ground state m_s_ = ±1 and transitions to the excited state, it decays to an unstable state and eventually relaxes back to the ground state without emitting fluorescence photons. Additionally, when the MW frequency matches the electron spin resonance transition frequency, the MW field excites the electron spin in the ground state of the NV centers to transition from the m_s_ = 0 state to the m_s_ = ±1 state, leading to a reduction in photoluminescence (PL) intensity [23,24]. Consequently, MW electromagnetic field information can be derived from the PL intensity of NV centers through ODMR. Moreover, MW power can be determined from the constant of the ODMR [25,26]. This renders the NV center sensitive to the magnetic field B and the zero-field splitting *D* [27,28], which is induced by the crystal field and varies with temperature, *D* = *D*(T), due to the thermal expansion and contraction of the lattice as a function of temperature T.

**Figure 2:**
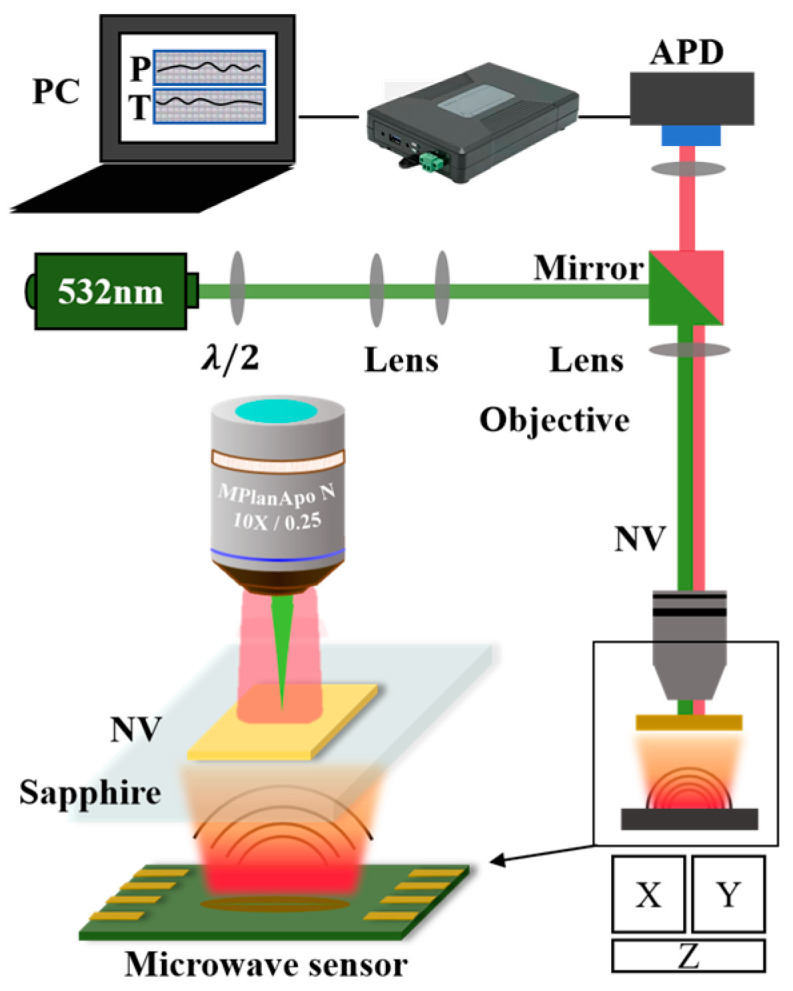
Schematic diagram of the system.

This system is a custom-built confocal optical setup, comprising an NV center sensing system and a fluorescence signal detection system. Here, all components are secured to a shock-absorbing optical platform (Omtools, HGZO2012D, Red Star Yang Technology, Wuhan, China). In the experiment, an IB-type diamond with dimensions of 5 × 5 × 0.3 mm^3^, cut along the [100] direction, was utilized. The diamond underwent 3 h of high-energy electron irradiation (with a dose and energy of 5.44 × 10^17^ cm^−3^ and 10.0 MeV, respectively) followed by 2 h of annealing at 850 °C, leading to the formation of vacancies near nitrogen atoms and the creation of stable NV centers [29]. As depicted in Figure 2, the NV center, serving as the sensing unit, is excited by a 532 nm laser. The resulting fluorescence is collected by an avalanche photodiode (APD) after passing through a lens assembly and then transmitted to the host computer for real-time signal analysis. The experimental samples included three microwave sensors of varying sizes and shapes, as shown in the physical images in Supporting Figure S1. The signals were generated by a microwave source (Rohde & Schwarz SMB-100B, Munich, Germany), and the strengths of the signals were magnified by a power amplifier and then fed into the patch antenna. Meanwhile, the microwave field, in the range of 2.82–2.92 GHz, transmitted into free space and was received by the NV centers in the diamond. The testing distance between the samples and the diamond was controlled using a three-axis high-precision translation stage. During testing, the APD and oscilloscope collected the ODMR signal in real time, providing output data on power and temperature.

## 3. Results

Through temperature control experiments utilizing a thermocouple thermometer and a heating stage, the ODMR contrast was systematically calibrated against the microwave power, and the *D*-value frequency shift was observed as a function of temperature. The temperature of the diamond was determined based on the shift in the ODMR spectrum, with a temperature change rate of about 96.5 mK/Hz, calibrated using a piezoelectric thermometer in contact with the diamond [30]. The thermocouples used were of the K type, diagonally connected to the upper surface of the diamond sample. Additionally, by summing the ODMR signals from the NV centers, the frequency shift was calculated. The resonance frequencies can be obtained by fitting the ODMR spectra using the Lorentzian function, and the formula is expressed as:

(2) $$y\left(f\right)=y_{0}+\frac{2}{\pi }\left(\frac{A\delta_{V}}{4{\left(f-f_{C}\right)}^{2}+{\delta_{V}}^{2}}\right)$$

where *y*_0_ is the fluorescence intensity in the non-resonant region, *A* represents the area of the resonance peak, *δ*_v_ is the full width at half maximum of the ODMR spectrum, while *f* and *f*_c_ denote the frequency of the fitted point and resonance frequency, respectively. Based on the calibration results, power and heating condition measurements were conducted for the three sensors, with the primary sensing units being three differently shaped microwave antennas.

**Figure 3:**
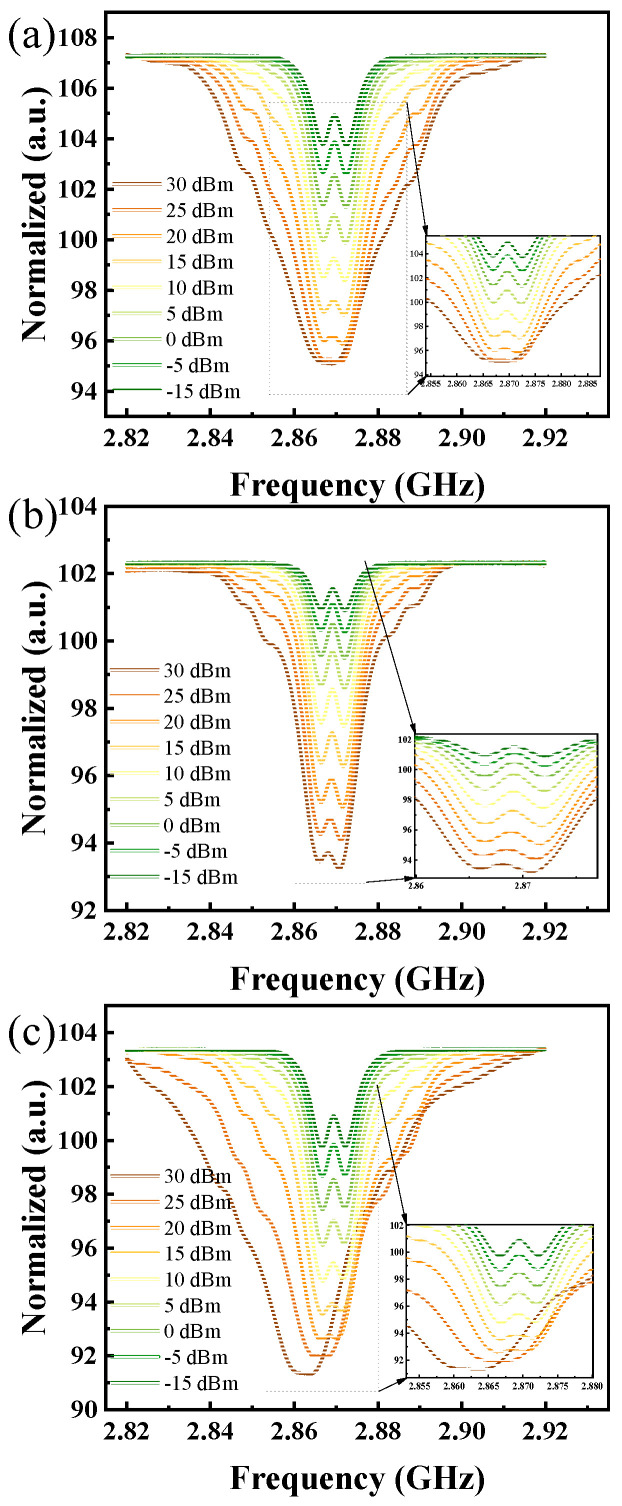
Test results of ODMR signals for samples as the input power varies. (**a**) Sensor 1, (**b**) Sensor 2, (**c**) Sensor 3.

By detecting the movement and distribution of spin levels, it is possible to simultaneously measure two different physical quantities. The results presented in Figure 3 are derived from experiments where different input powers were tested, using this detection system to evaluate the microwave radiation performance of three differently shaped microwave sensors at the same frequency. The ODMR spectra, as illustrated in the figure, demonstrate significant differences in the test outcomes among the three sensors. This variation is strongly linked to the sensor structure. As seen in the inset figure, notable changes in contrast and zero-field splitting (D) are evident. Based on this, the variations in power and temperature of the microwave sensors can be clearly observed. The effects of these two physical quantities do not interfere with each other, providing a viable basis for real-time synchronized output.

**Figure 4:**
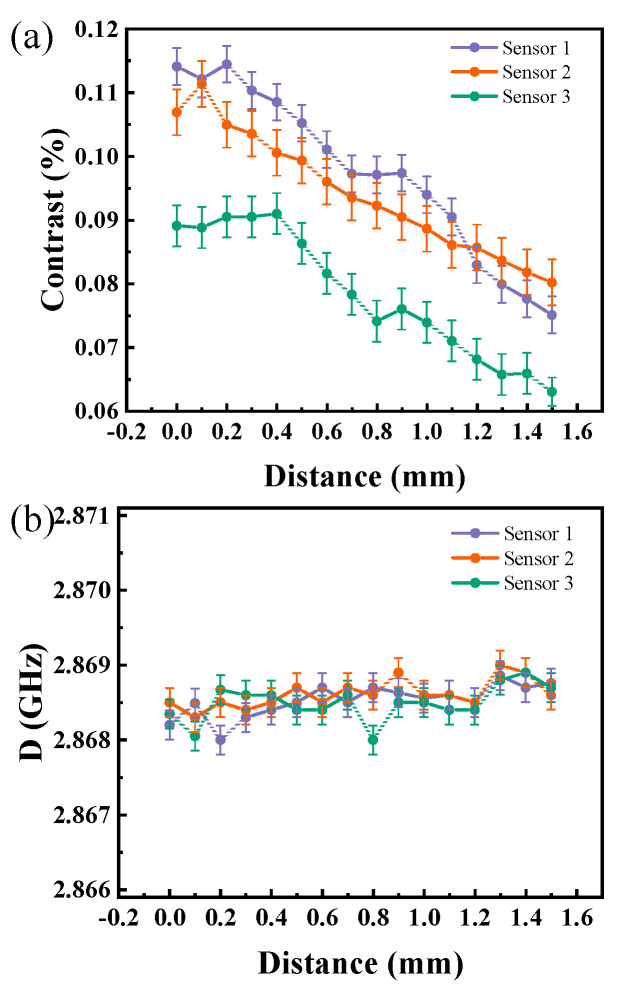
(**a**) The contrast variation of microwave sensors at different testing distances. (**b**) The frequency shift variation of D values at different distances.

Data extraction and analysis enable the identification of variations in radiation power and temperature across three types of sensors at varying excitation powers, as depicted in Figure 4a. Upon analysis, it is observed that Sensor 1 exhibits the highest maximum contrast, while Sensor 3 displays the lowest. As the microwave power opens, attenuation increases with distance. Due to thermal radiation, the impact of temperature on the results is subdued in short-range tests. As demonstrated in Figure 4b, temperature exhibits minimal fluctuations within a 1 mm range. Utilizing a noncontact detection approach, this system captures temperature and power data from a sample’s surface. Consequently, it is suitable for application in sensor detection within industrial packaging.

**Figure 5:**
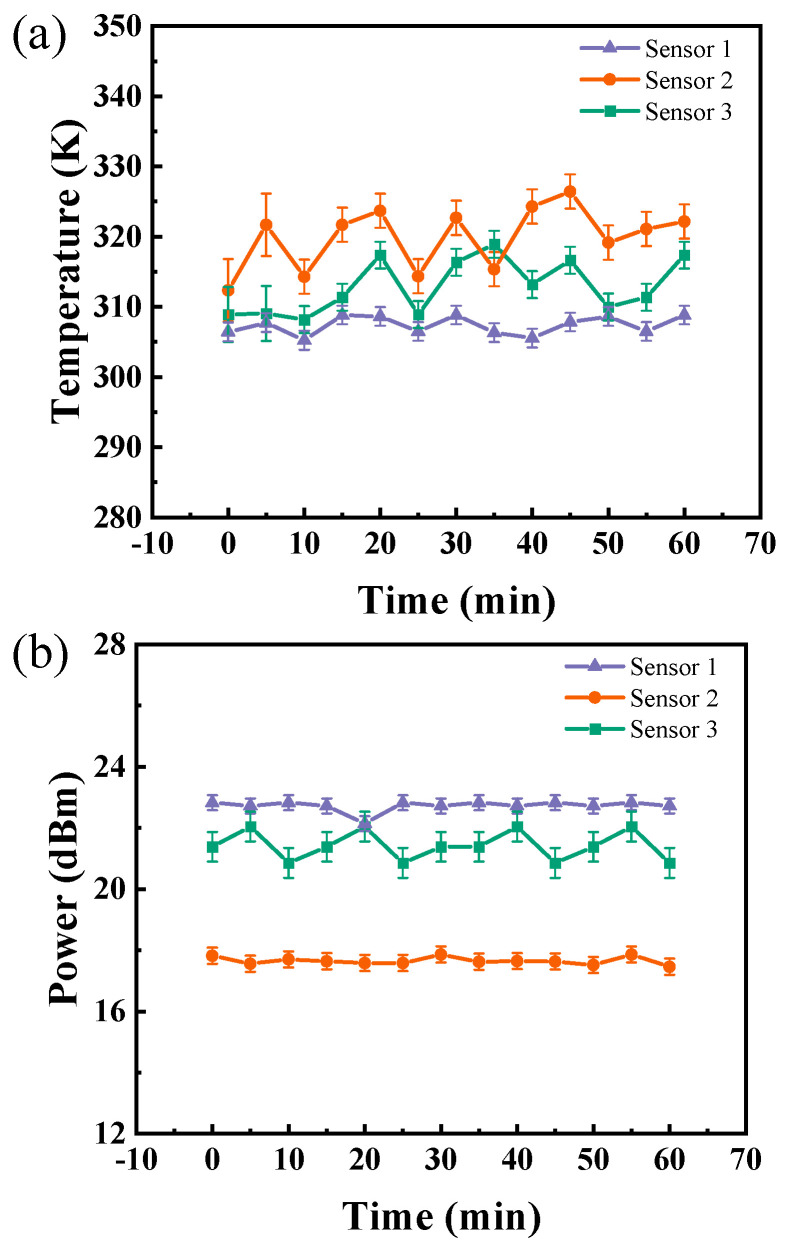
(**a**) The variation in radiation temperature over time for the three sensors. (**b**) The variation in contrast over time for the three sensors.

The real-time temperature and contrast of the sensors were recorded over one hour. Utilizing a data acquisition card for real-time data collection, the trends in temperature and power variations across different samples over time were documented. Figure 5a illustrates that Sensor 1 exhibits the least variation in temperature, signifying its stable performance over time. Sensor 2 displays moderate temperature fluctuations, while Sensor 3 experiences significant variations, indicating greater instability and increased heating over time. Figure 5b reveals that the power intensity of Sensor 1 remains relatively stable in comparison with that of Sensor 3. Although the stability of Sensor 2 is also noted, its radiation power is not comparable with that of Sensor 1. These observations confirm that Sensor 1 surpasses the other sensors in terms of temperature stability and consistent power output over time. Sensor 2 achieves moderate performance, whereas Sensor 3 demonstrates the greatest instability in both temperature and power output, establishing Sensor 1 as the most dependable option for applications necessitating stable performance and minimal heating over time. This detection method provides an intuitive assessment of sensor performance and enables precise long-term monitoring of heating parameters during sensor production and testing.

**Figure 6:**
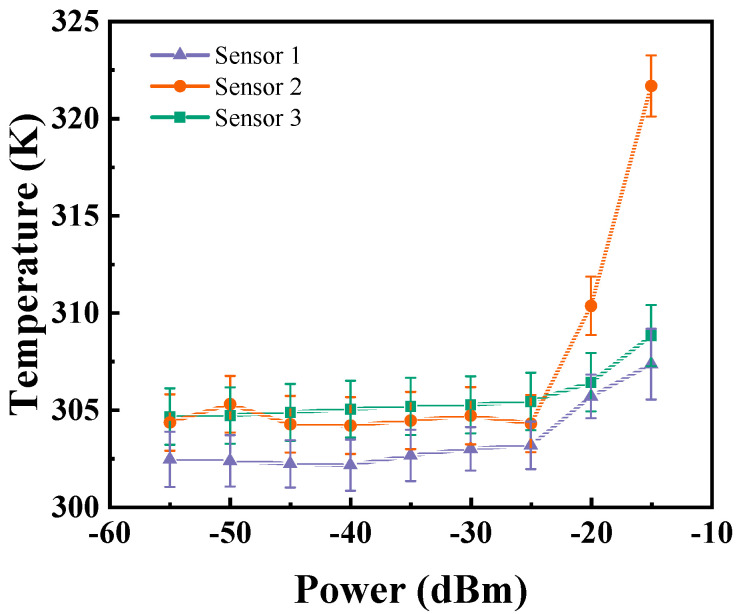
Effects of different input powers on sensor heating.

Based on the results presented, significant performance differences are observed among the three sensors when subjected to microwave signals at a uniform frequency, as depicted in Figure 6. This graph illustrates the correlation between microwave input power and the ODMR contrast for the three distinct sensors. The performances of Sensor 1 (orange circles), Sensor 2 (blue triangles), and Sensor 3 (green squares) are compared across various input power levels. Sensors 1 and 2 maintain relatively stable ODMR contrast as the input power increases, demonstrating their robustness under fluctuating power conditions. In contrast, Sensor 3 exhibits a marked decrease in ODMR contrast with increasing input power, indicative of a significant performance decline at higher power levels. Specifically, at the maximum input power levels, the contrast for Sensor 3 decreases sharply, a pattern not evident in the other two sensors.

The analysis demonstrates that Sensors 1 and 2 are more appropriate for applications that demand consistent performance across various input power levels. Sensor 3, due to its pronounced decline in performance under higher power conditions, may be less suitable for applications that require high power input. This comparative evaluation underscores the different sensitivities of each sensor type to input power, with Sensor 3 being the most negatively impacted by increased power levels.

**Figure 7:**
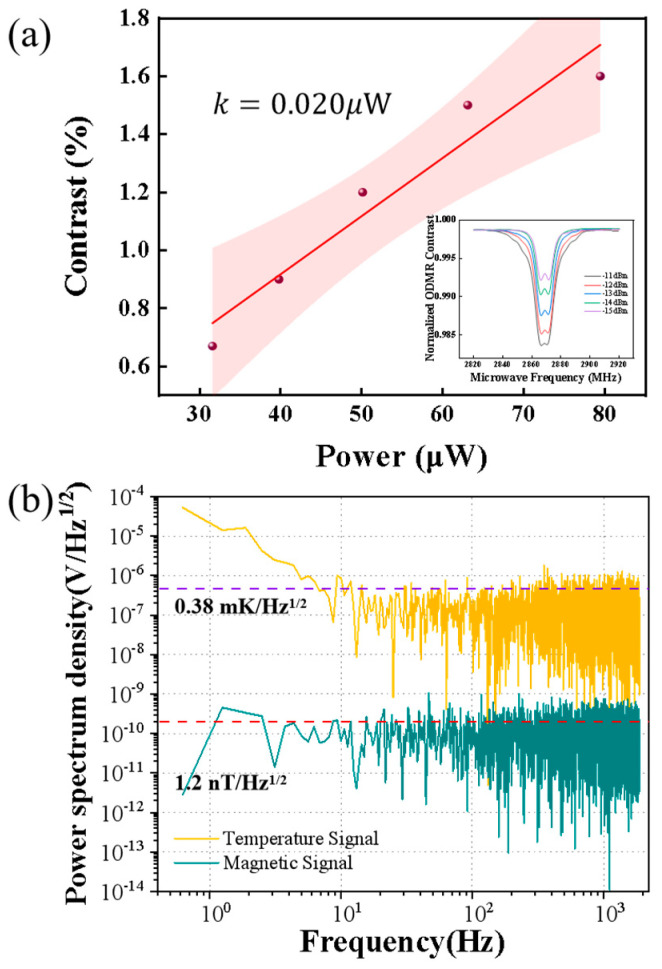
(**a**) The relation between contrast and microwave power. (**b**) The power spectral density plot of the system, where yellow represents magnetic sensitivity and green represents power spectral density.

Finally, we evaluated the accuracy of the system during power testing. We tested the contrast of the microwave power when it was very weak, as shown in Figure 7a. The power resolution of the system is established at 0.01 dBm. The minimum resolution of microwave power is 20 nW. The illustration shows the contrast of microwave power at −11 dBm to −15 dBm. The testing power range can span from tens of microwatts to several hundred watts, which generally covers most common microwave sensors. The fluorescence noise spectra were measured. Fourier transform was applied to the collected fluorescence intensity. Subsequently, the magnetic noise density spectrum at different sampling frequencies was calculated using the aforementioned equation. Through analysis of the noise spectrum of the signal, it was found to be sensitive to both magnetic field and temperature, as illustrated in Figure 7b.

By analyzing the power spectral density of the measured signal, the sensitivity of the system to the microwave magnetic field and temperature is obtained, with results presented in Figure 7. The detection sensitivity of the magnetic field at the NV centers is defined as the smallest microwave magnetic field detectable at a signal-to-noise ratio of 2 [31]. The corresponding formula is provided as follows [32]:

(3) $$S_{B}\approx \frac{P_{L}}{\gamma_{e}}\cdot \frac{\tau \sqrt{hc}}{C\sqrt{P_{f1}\lambda }}$$

where *P*_L_ ≈ 0.707 is the Lorentzian line shape parameter, *h* indicates the Planck constant, *P_f_*_1_ is the collected red fluorescence power, λ ≈ 637 nm is the wavelength, and c represents the speed of light in a vacuum. *τ* is the full width at half maximum (FWHM) of the ODMR spectrum, and *C* indicates the fluorescence contrast. The microwave field detection sensitivity of the system is calculated to be 1.2 nT/Hz^1/2^.

For the temperature measurement, sensitivity is calculated in a manner akin to that used for microwave magnetic field detection, though the spectral drift parameter shifts from the spin ratio to the temperature drift parameter. Consequently, the temperature detection sensitivity is determined to be 0.38 mK/Hz^1/2^.

## 4. Conclusions

This paper presents a real-time temperature and power detection system that utilizes NV centers, examining the effects of distance, input power, and time on temperature and output power. This study illustrates the system’s capability for contactless real-time testing. It is noted that the impact of identical input power levels on the output power and temperature varies markedly among different microwave sensors, a factor crucial for rapidly assessing microwave device performance and selecting high-quality sensors. Real-time monitoring of sensor radiation power and heating provides vital data for sensor development and testing. The magnetic sensitivity of the NV magnetometer is about 1.2 nT/Hz^1/2^, and the temperature sensitivity of the temper is about 0.38 mK/Hz^1/2^. This research further supports the advancement and miniaturization of integrated NV multiphysics detection systems. Future efforts will focus on enhancing detector compactness and improving fluorescence collection efficiency to increase the sensitivity of measurements for microwave power and temperature. At the same time, the detection bandwidth will be further expanded by means of an applied magnetic field.

## Data Availability

The original contributions presented in the study are included in the article, further inquiries can be directed to the corresponding author.

## Supplementary Materials

The following supporting information can be downloaded at: https://www.mdpi.com/article/10.3390/mi15111305/s1, Figure S1: The picture of microwave sensors.
