# Supplementary material for: Simultaneously Detecting the Power and Temperature of a Microwave Sensor via the Quantum Technique
Source: Micromachines (Basel). 2024 Oct 28;15(11):1305. doi: 10.3390/mi15111305 (PMC11596659; doi:10.3390/mi15111305)
Supplement: Supplementary file 1 [file micromachines-15-01305-s001.zip › micromachines-3205301-supplementary.pdf]

micromachines

Supplementary Materials

Simultaneously Detecting the Power and Temperature of a Microwave Sensor via the Quantum Technique

Zhenrong Zhang, Yuchong Jin, Jun Tang and Jun Liu

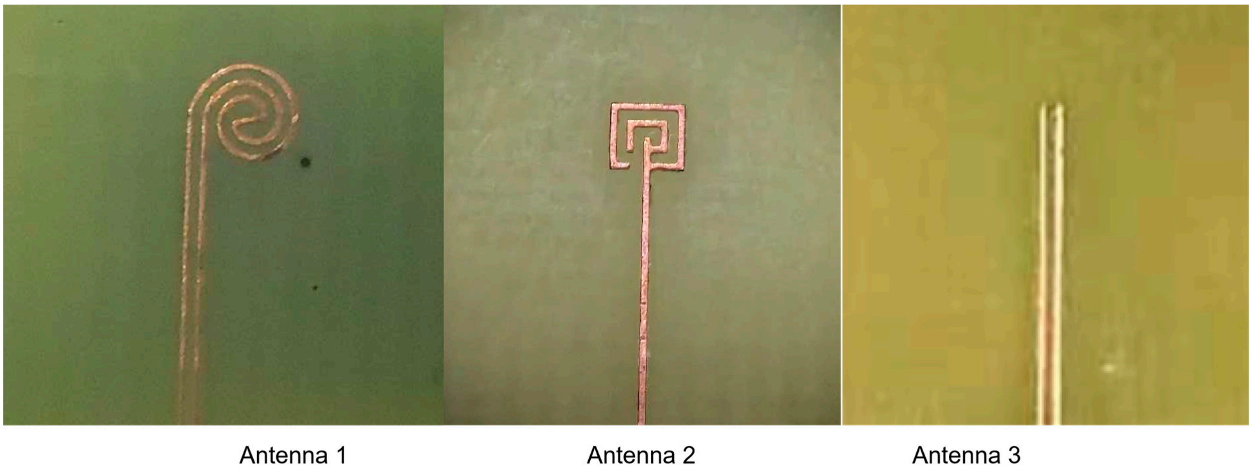

Figure S1. The picture of microwave sensors.
